# Advancing Machine Learning Strategies for Power Consumption-Based IoT Botnet Detection

**DOI:** 10.3390/s25247553

**Published:** 2025-12-12

**Authors:** Almustapha A. Wakili, Saugat Guni, Sabbir Ahmed Khan, Wei Yu, Woosub Jung

**Affiliations:** Department of Computer and Information Sciences, Towson University, Towson, MD 21252, USA; awakili1@students.towson.edu (A.A.W.);

**Keywords:** Internet of Things (IoT), botnet detection, power consumption, deep learning, hybrid models, machine learning, CHASE’19 dataset

## Abstract

The proliferation of Internet of Things (IoT) devices has amplified botnet risks, while traditional network-based intrusion detection systems (IDSs) struggle under encrypted and/or sparse traffic. Power consumption offers an effective side channel for device-level detection. Yet, prior studies typically focus on a single model family (often a convolutional neural network (CNN)) and rarely assess generalization across devices or compare broader model classes. In this paper, we conduct unified benchmarking and comparison of classical (SVM and RF), deep (CNN, LSTM, and 1D Transformer), and hybrid (CNN + LSTM, CNN + Transformer, and CNN + RF) models on the CHASE’19 dataset and a newly curated three-class botnet dataset, using consistent preprocessing and evaluation across single- and cross-device settings, reporting both accuracy and efficiency (latency and throughput). Experimental results demonstrate that Random Forest achieves the highest single-device accuracy (99.43% on the Voice Assistant with Seed 42), while CNN + Transformer shows a strong accuracy–efficiency trade-off in cross-device scenarios (94.02% accuracy on the combined dataset at ∼60,000 samples/s when using the best-performing Seed 42). These results offer practical guidance for selecting models under accuracy, latency, and throughput constraints and establish a reproducible baseline for power-side-channel IDSs.

## 1. Introduction

Intrusion Detection Systems (IDSs) have evolved significantly from rule-based to data-driven mechanisms, propelled by the rapid growth of cyber threats across connected digital infrastructures. Traditional IDSs relied heavily on signature-based detection, making them insufficient against novel or obfuscated attacks [1,2,3,4,5,6]. As systems became more complex, researchers began applying machine learning (ML) and deep learning (DL) techniques to IDSs, offering dynamic threat modeling and adaptive anomaly detection capabilities [2,7,8,9,10,11,12]. The significance of generic predictive designs is further highlighted by their successful application in scalable IoT management and intelligent transportation systems [13,14]. However, these approaches encounter new limitations with the proliferation of Internet of Things (IoT) devices, which are often resource-constrained and operate under minimalistic communication paradigms across heterogeneous platforms, making conventional IDS deployment and traffic monitoring increasingly difficult [15,16,17,18,19].

A particularly concerning threat vector in the IoT ecosystem is the botnet—networks of compromised devices that can be remotely orchestrated to launch various large-scale attacks such as distributed denial of service (DDoS), data exfiltration, and credential stuffing, among others. While most existing detection systems rely on network traffic analysis, their efficacy diminishes significantly in environments with encrypted, obfuscated, or low-volume communications. This observation aligns with studies on the limitations of flow-based analysis for stealthy or idle botnets [20,21]. As an alternative, recent efforts have explored side-channel signals such as power consumption, which offer device-level behavioral signatures that are persistent, hard to spoof, and independent of network visibility [22].

Along this promising paradigm, Jung et al. [23] were among the first to model this paradigm, introducing a convolutional neural network (CNN)-based classifier trained on power profiles collected from real IoT devices. Using the CHASE’19 dataset [23], their study reported high accuracy and generalizability across device types and botnet variants. Their framework laid the groundwork for subsequent power-based detection systems and motivates the direction of our research. Following this foundational work, several studies have explored alternative architectures, hardware platforms, and deployment mechanisms. For example, DeepAuditor [24] extended this idea by developing a distributed CNN-based online IDS using power signals. Still, it was limited to a single model type (i.e., CNN) and did not benchmark generalization performance across device types. DeepShield [25] introduced a privacy-preserving lightweight framework using knowledge distillation, yet focused solely on inference efficiency without exploring trade-offs across other model families. The demo version [26] emphasized system architecture rather than model evaluation. Furthermore, Li et al. [27] proposed a smart plug design to support real-time detection, but lacked comparison across ML/DL architectures or discussion of generalization performance. On the classical machine learning side, Ma et al. [28] proposed a handcrafted statistical feature extraction pipeline with an SVM classifier. While efficient, this method does not capture the temporal dependencies inherent in IoT signals. Similarly, Przybocki et al. [29] analyzed power draw characteristics under attack scenarios but did not integrate machine learning into the detection pipeline.

In contrast to these architecture-specific works, our study presents a unified, comparative framework that evaluates classical, deep, and hybrid models across various domains. Specifically, we use consistent preprocessing and evaluation protocols on commonly-used datasets [23,30] to assess both classification accuracy and computational efficiency across single- and cross-device settings. Specifically, we investigate: (i) Classical models: Support Vector Machine (SVM) and Random Forest (RF), (ii) Deep models: CNN, Long Short-Term Memory (LSTM), and a 1D Transformer, and (iii) Hybrid architectures: CNN + LSTM, CNN + Transformer, and CNN + RF.

These models are evaluated under consistent experimental protocols using a single device, and cross-device validation methods are employed to ensure consistency. Our findings suggest that deep learning models achieve high accuracy in homogeneous settings, but their performance often degrades in cross-device scenarios. This broad benchmarking not only quantifies the trade-offs of each model class but also identifies architectures, such as CNN + Transformer, which balance accuracy, generalizability, and real-time viability, key factors for practical IoT botnet detection at the network edge. To the best of our knowledge, this work presents the first comprehensive evaluation of classical, deep, and hybrid machine learning architectures for power-based IoT botnet detection. Our framework highlights key trade-offs in generalization and computational efficiency across heterogeneous devices, with a focus on real-world applicability for edge deployments.

**Figure 1:**
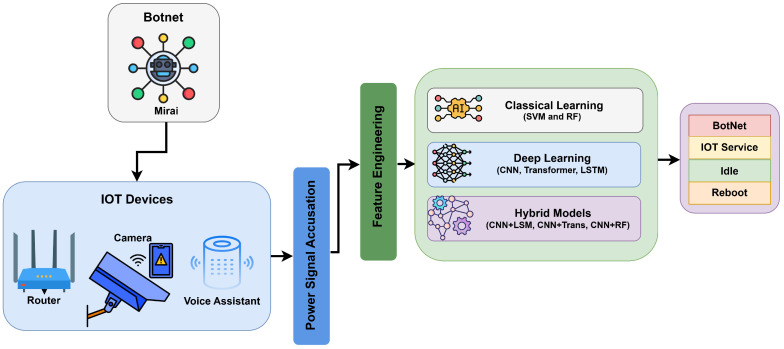
Overview of the proposed IoT botnet detection framework using power signal analysis. The system captures power consumption from IoT devices (e.g., routers, cameras, voice assistants), extracts features, and applies classical, deep, or hybrid learning models to classify device behavior into Botnet, Idle, Reboot, or Normal IoT Service states.

An overview of the complete power-based IoT botnet detection pipeline used in this study is shown in Figure 1. The figure summarizes how power signals are captured from IoT devices, preprocessed, and analyzed using classical, deep, and hybrid learning models to classify behaviors such as Botnet, Idle, Reboot, and Normal IoT Service.

This work is a domain-specific, unified benchmarking study rather than a proposal of new neural blocks. The key contributions of this paper are summarized as follows:

- A reproducible pipeline that compares classical (SVM, RF), deep (CNN, LSTM, 1D Transformer), and hybrid (CNN + LSTM, CNN + Transformer, CNN + RF) models on power side-channel signals under single-device, cross-device, and leave-one-device-out (LODO) regimes.
- A generalization-focused evaluation that makes device shift a first-class concern and quantifies when accuracy drops across devices.
- A deployment-oriented analysis that reports accuracy, together with latency and throughput, to guide edge implementations.
- Evidence indicates that RF attains the highest single-device accuracy on CHASE’19 99.43% on the Voice Assistant device with the best seed, while CNN + Transformer provides the best accuracy–efficiency trade-off in cross-device settings 97.83% test accuracy for the best seed (Seed 43) with a throughput of approximately 48,000 samples/s.

In short, there is a critical need for more rigorous and reproducible benchmarking to uncover trade-offs among accuracy, efficiency, and generalizability. By sharing our comprehensive evaluation pipeline and highlighting gaps in prior work, we aim to inspire the broader research community to further explore hybrid models, refine cross-device validation strategies, and design resource-efficient IDS solutions tailored for edge deployment in heterogeneous IoT environments.

The remainder of the paper is structured as follows. Section 2 presents the background of IDS technologies, the limitations of network traffic-based detection in IoT, and recent advances in power-based machine learning models. Section 3 describes the datasets, preprocessing techniques, and architectural designs of each model. Section 4 details the experimental setup, presents the results, and discusses and interprets key findings and deployment implications. Section 6 outlines several directions of further research and Section 7 concludes the paper.

## 2. Background and Related Works

### 2.1. From Signature to Learning-Based IDS in IoT

IDS is a defense mechanism for identifying unauthorized activity within digital environments. Classical IDS approaches are predominantly signature-based, relying on known patterns to flag suspicious behavior. While effective against previously observed threats, these systems struggle to detect zero-day exploits and sophisticated obfuscation techniques [1]. Researchers have increasingly applied anomaly-based detection and machine learning/deep learning techniques to overcome these limitations, which model normal system behavior and flag deviations as potential threats [2,31].

Recent surveys reiterate the trade-offs between signature and anomaly detection: signature (misuse) methods achieve low false-alarm rates but miss unknown attacks and require constant rule maintenance, whereas anomaly methods generalize to unseen behaviors but tend to raise more false positives and depend on a robust definition of “normal” [32]. In IoT, traditional IDSs designed for conventional networks struggle with device and protocol heterogeneity, zero-day attacks, scalability, and the overhead of manual signature updates, which motivates machine-learning-based IDSs that can adapt to evolving traffic patterns [32]. The importance of balancing efficiency and robustness has been observed in broader digital security domains, including decentralized smart applications [33], metaverse scam detection [34], and online fraud analysis [35]. These constraints undermine the effectiveness of traditional IDSs. Similarly, Liu and Lang [2] provided a broad survey of learning techniques for IDSs, covering feature extraction, supervised and unsupervised models, and evaluation practices. These methods have shown significant promise in conventional networks but face new challenges in IoT environments. As highlighted by Hajiheidari et al. [18], IoT infrastructures exhibit high heterogeneity in hardware, operating systems, and communication protocols, compounded by limited processing power and memory. A contemporary review highlights recurring challenges for IoT IDSs, including class imbalance, feature selection under resource constraints, model complexity, and the need to adapt to evolving attack vectors; lightweight on-device models and careful efficiency reporting are repeatedly recommended [36]. This study also emphasizes standardized metrics and real-world testing, noting gaps in outdated, non-IoT-specific datasets and the importance of measuring training and inference time, as well as resource utilization [36].

These constraints undermine the effectiveness of traditional IDSs, particularly those that rely on deep packet inspection or heavy network logging [16]. While deep learning has uncovered useful patterns for intrusion detection, recent analyses caution that real-world performance, false-positive rates, explainability, and operational deployment across cloud, edge, and IoT remain open challenges. Closing the gap between results in controlled settings and outcomes in production environments is a key direction for the next few years [37].

### 2.2. IoT Botnets: Threats and Traffic-Centric Detection

Botnet attacks remain one of the most pervasive threats in IoT, capable of leveraging thousands of devices for malicious purposes. Several studies have proposed machine learning-based models that rely on traffic features or metadata for botnet detection. For example, Bagui et al. [38] demonstrated the efficacy of SVM and RF classifiers on real-world botnet datasets. Idrissi et al. [39] introduced BotIDS, a CNN-based detection system that outperformed LSTM and GRU models. Likewise, Singh et al. [40] employed the LOBO-CV technique to evaluate generalization on unseen botnet families. Complementing these results, Ullah et al. presented a multi-model framework spanning BOT-IOT, CICIOT2023, and IoT-23, reporting 100%/99.2%/91.5% accuracy respectively and discussing efficiency and class-imbalance challenges on IoT-23 [41].

In addition, Rodríguez-Gómez et al. [20] offered a lifecycle-oriented taxonomy of botnets, including infection, propagation, and command-and-control (C&C) phases. Their framework offers a conceptual foundation for understanding where and how detection can be applied. Recent deep-learning IDS baselines also report near-state-of-the-art performance on modern IoT traffic. For example, Hossain showed 1D-CNN reaching 99.12% (multi-class) and 99.53% (binary) on CIC IoT-DIAD 2024 while emphasizing scalability for real-time detection [42]. Furthermore, Alrawi et al. [21] performed a large-scale analysis of IoT malware behavior, reinforcing the need for detection methods that operate independently of network visibility. Targeting early-stage botnet activity, Korba et al. develop a semi-supervised, explainable anomaly-detection approach that detects stealthy C&C traffic on IoT-23 with a 99.51% detection rate and 1.09% false positives, illustrating traffic-centric detection that does not require labeled attacks [43].

### 2.3. Power Side-Channel for Device-Level Detection

Due to the increasing use of encryption and stealth tactics, power consumption has emerged as a compelling alternative for device-level behavioral monitoring. As continuous and difficult to manipulate, power signals serve as side channels for modeling device activity [19,22]. To this end, Jung et al. [23] showed that CNNs trained on power data from the CHASE’19 dataset can distinguish benign from compromised states with over 96% accuracy. Ding et al. [44] advanced this line of work by employing sequence-to-sequence modeling in DeepPower. Also, Tekin et al. [45] compared edge, cloud, and TinyML deployments, highlighting trade-offs in inference time and energy consumption. Building on these early power-based intrusion detection efforts, Campos et al. [46] proposed an intrusion detection framework that fuses side-channel power measurements with machine learning classifiers for general IoT environments, further demonstrating that power traces can discriminate benign and malicious activity. Recent work also confirms that power and energy consumption can sustain practical IoT intrusion and anomaly detectors in constrained edge and 6G ready settings, which motivates our focus on this modality [47,48]. A recent study finds that power is a non-intrusive, out-of-band signal that is hard to tamper with, and it surveys detection pipelines from feature-based to deep learning methods [49].

In smart home scenarios, Nimmy et al. [50] used Raspberry Pi data to train a deep feedforward neural network, achieving 99.2% accuracy in detecting anomalies. Beyond smart home and gateway devices, side-channel IDS was also prototyped for mission-critical UAVs, reporting ROC up to 0.995 and accuracy near 98% when detecting hardware Trojans from power telemetry [51]. Lightbody et al. [52] further presented a side-channel power acquisition framework for IoT devices and showed that common attacks induce measurable variations in current draw that can be captured in reusable datasets for intrusion detection. Merlino and Allegra [22] surveyed various power-based detection methods and emphasized the underutilized potential of such signals in IoT cybersecurity. Complementing this survey, Dragon-Pi releases labeled power traces from DragonBoard 410c and Raspberry Pi testbeds and introduces an unsupervised convolutional autoencoder baseline for anomaly detection [53].

To further improve model efficiency, Saied et al. [19] explored filtering-based feature selection techniques for IoT botnet detection, and Saied et al. [31] advocated for explainable AI in IDSs to increase transparency and trust in ML models. Albasir et al. also argued [54] that AI-driven monitoring of resource and energy usage is a promising avenue for protecting IoT and cyber-physical devices, reinforcing the relevance of power- and energy-aware intrusion detection. DeepAuditor [24] applied a CNN-based model for distributed intrusion detection using power side-channel signals, achieving strong detection performance. However, it did not benchmark generalization across devices or evaluate alternative architectures. Recent work also applied deep learning directly to side-channel leakage to flag malicious activity at the hardware level, reinforcing the trend toward data-driven power analysis [55]. DeepShield [25] introduced a lightweight, privacy-preserving framework using compressed CNNs and knowledge distillation to enable split inference between IoT devices and local gateways. It reached 96.3% accuracy while reducing model size by 25×, yet focused only on a single architecture without considering broader model diversity.

Further, Ma et al. [28] proposed a statistical feature-based SVM classifier, which offers interpretability and low overhead but lacks temporal modeling and scalability to dynamic behaviors. Li et al. [27] investigated a smart plug for local malware detection, combining hardware monitoring with deep feedforward networks. Still, they did not evaluate cross-device robustness or compare it with alternative model types. Przybocki et al. [29] performed a fine-grained analysis of power draw characteristics in physical and virtual devices under attack, but did not apply or test machine learning methods. Lastly, the demo version of DeepAuditor [26] highlighted the system’s architectural deployment but omitted quantitative performance metrics comparisons.

### 2.4. Gap Analysis and Positioning

To summarize, these existing studies underscore the growing interest in power-based IDSs, but also reveal limitations, such as reliance on specific models, a lack of hybrid or classical baselines, or the absence of cross-device evaluation. Our work addresses these gaps by offering a comparative evaluation across diverse model families under unified preprocessing, sampling, and validation protocols. Recent surveys and empirical studies further underline persistent deployment challenges in IoT IDSs (e.g., class imbalance, drift, and operational constraints) and the need for standardized, reproducible evaluation [32,36,37]. Traffic-centric botnet detectors report strong headline accuracy on modern corpora, yet generalization remains uneven across BOT-IOT, CICIOT2023, and IoT–23 [41,42,43]. In parallel, side-channel security has gained momentum with systematization and new datasets, but comparisons rarely span multiple modeling families under a unified protocol or report deployment metrics [49,51,53,55]. Accordingly, the open gap is a reproducible, domain-specific comparison across classical, deep, and hybrid models that centers on device-shift generalization and edge feasibility. Our work addresses this gap by standardizing preprocessing, splits, and metrics, reporting accuracy alongside latency and throughput, and including leave-one-device-out evaluations to approximate device shift, with evaluations on CHASE’19 [23] and the three-class botnet dataset [30] under consistent protocols.

We choose CHASE’19 because it provides high-rate (5 kHz) power traces segmented into fixed 1.5 s windows (7500 samples) with labels for four device states, allowing consistent single-device, cross-device, and leave-one-device-out evaluations within the same corpus [23]. It is widely referenced in the power-based IoT IDS studies we cite, thereby preserving comparability with prior work. We therefore position our results as a baseline benchmark and plan to extend the pipeline to newer datasets in future work.

## 3. Our Approach

This section describes the overall processing pipeline for power consumption–based IoT botnet detection. Our approach encompasses data acquisition and preprocessing, classical feature engineering, and the design and training of several models, ranging from classical machine learning to deep learning and hybrid architectures.

**Figure 2:**
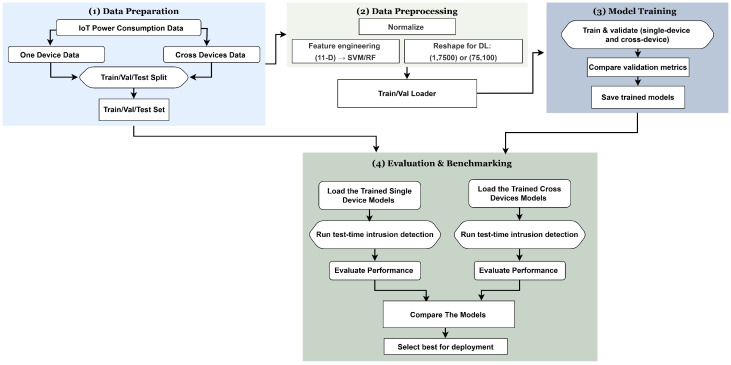
Workflow for power-based IoT botnet detection across four stages: data preparation, preprocessing, model training, and evaluation/benchmarking.

As shown in Figure 2, the pipeline proceeds through four stages:

- Stage 1. Data Preparation: IoT power traces are grouped into one-device and cross-device sets and split into Train/Val/Test.
- Stage 2. Data Preprocessing: Each 1.5 s window (7500 samples) is normalized and then routed either to (i) feature engineering, an 11-D vector of time/frequency statistics plus low-band energy used by SVM/RF or to (ii) deep-learning reshaping: (1,7500) for CNN/CNN + RF and (75,100) for LSTM, a 1D Transformer, and hybrid models; Train/Val loaders are then built.
- Stage 3. Model Training: We train and validate under both single-device and cross-device regimes, compare validation metrics, and save the checkpoints.
- Stage 4. Evaluation and Benchmarking: The trained single-device and cross-device models are loaded in parallel lanes to run test-time intrusion detection (with the same normalization), compute accuracy, precision, recall, F1, ROC–AUC, and efficiency (latency, throughput), after which we compare models and select the best configuration for deployment.

### 3.1. Workflow

#### 3.1.1. Dataset and Preprocessing

We adopt the CHASE’19 dataset, which consists of power consumption measurements sampled at 5 kHz. Each instance is constructed from a 1.5 s window and contains 7500 data points. The dataset is divided into four classes that capture distinct operational and anomalous patterns: (i) IoT Service (0) represents normal device operations, (ii) Reboot (1) captures power profiles during device restarts, (iii) Idle (2) reflects baseline power usage without activity, and (iv) Botnet (3) contains signatures produced by botnet-induced anomalies.

Balanced datasets are generated for each device type (e.g., routers, cameras, voice assistants) with 2200 instances per class. The individual datasets are concatenated for cross-device experiments, resulting in a combined dataset of 26,400 windows of length 7500 samples.

##### Three-Class Botnet Dataset (Robustness Benchmark)

To evaluate model robustness, we additionally employ the Three-Class Botnet Dataset [30] constructed from various IoT power traces. The classes are defined as follows: (i) Idle (0) represents idle traffic with minimal device activity, (ii) Benign (1) captures traffic from regular non-malicious IoT usage, and (iii) Infected (2) aggregates traffic from various botnet-infected scenarios (e.g., Mirai, Moobot, Satori, Okiru, Sora). To avoid optimistic bias, normalization parameters were fit on the training split only, and splits were created to prevent overlap between recordings across train/val/test.

The original dataset contained a highly imbalanced distribution. To ensure a strictly fair evaluation and eliminate majority class bias, we performed rigorous deduplication and applied random stratified sampling. The resulting dataset is perfectly balanced, containing exactly 3000 instances for each of the three classes (Idle, Benign, Infected), for a total of 9000 samples. A strict data leakage check was implemented to ensure zero overlap between training and testing sets based on row-level byte hashing.

#### 3.1.2. Preprocessing and Feature Engineering

The preprocessing pipeline differs for classical and deep learning approaches.

##### Features for Classical Machine Learning

For traditional models, including Random Forest (RF) and Support Vector Machines (SVM), an 11-dimensional feature vector is extracted from each power signal. This feature vector comprises (i) Time-Domain Statistics: Mean, standard deviation, minimum, maximum, median, skewness, and kurtosis; (ii) Frequency-Domain Features: Mean, standard deviation, and maximum magnitude obtained from the Fast Fourier Transform (FFT); (iii) Band Energy: Energy in the low-frequency band (0–50 Hz).

##### Preprocessing for Deep Learning

For deep learning–based models, the following preprocessing steps are applied: (i) Normalization: Each raw signal is normalized to have zero mean and unit variance. (ii) Reshaping: For sequential models (e.g., LSTM, Transformer, and hybrids), each 7500-point signal is reshaped into a 75×100 matrix. For direct CNN models and the CNN + RF pipeline, signals are reshaped into a one-dimensional tensor of shape (1,7500).

### 3.2. Experimental Setup

All experiments were executed on a Lambda server with an Intel Core i9 CPU, 64 GB RAM, and two NVIDIA RTX 3080 GPUs (12 GB each), running Ubuntu 20.04 with PyTorch 1.13. We trained each deep and hybrid model using three random seeds (42, 43, and 44) and performed *k*-fold cross-validation on the training set, reporting the best-seed test accuracy and macro-F1 together with the mean ± standard deviation across folds. Since micro-averaged F1 coincides with overall accuracy in our multi-class setting, the reported test accuracy can be interpreted as the primary micro-weighted metric, while macro-F1 summarizes per-class balance. Training and inference were performed on NVIDIA RTX 3080 GPU (NVIDIA Corporation, Santa Clara, CA, USA) unless noted. As a reference for reproducibility, the key model and training hyperparameters are summarized in Table 1.

### 3.3. Model Architecture

We consider diverse models to benchmark classification performance and computational efficiency. We use the term classical ML for RF and SVM, deep for CNN, LSTM, and 1D Transformer, and hybrid for CNN + LSTM, CNN + Transformer, and CNN + RF. We avoid mixing with shallow to reduce ambiguity.

#### 3.3.1. Traditional Models

We consider the following classical models for traditional machine learning:

- Random Forest (RF): The RF classifier is trained on the engineered 11-dimensional feature vectors using 100 trees with a maximum depth of 20. These hyperparameters are selected to strike a balance between model complexity and interpretability, ensuring adequate ensemble diversity without overfitting on limited-dimensional inputs.
- SVM Pipeline: The SVM pipeline first extracts the 11-dimensional feature vector (using time-domain and frequency-domain statistics) from each raw signal. Following an 80/20 train-test split, an SVM with an RBF kernel is trained (with grid search tuning yielding parameters such as C=1 and γ=0.01). Although grid search improves performance slightly, the SVM achieves only about 78% accuracy on cross-device evaluation.

#### 3.3.2. Deep Learning Models

We consider the following deep learning models:

- Baseline CNN: A one-dimensional CNN is applied to inputs reshaped as (17, 500). The network uses 10 filters with a kernel size of 512 and a stride of 128, which is then followed by batch normalization, ReLU activation, and max pooling (kernel size of 4, stride of 4). Fully connected layers classify the flattened representation. Training is performed with Adam (learning rate of 0.001) over 20 epochs. Latency/throughput are reported in the Results tables.
- LSTM Model: Signals, reshaped to (75, 100), are processed by an LSTM with a hidden size of 128. The final hidden state is fed into a fully connected layer to yield the classification logits. Training uses Adam (learning rate of 0.001) over 20 epochs, with an average inference time of 0.0001710 s/batch.
- 1D Transformer Model: Inputs reshaped to (75, 100) are first projected via a linear embedding layer into a 128-dimensional space. Sinusoidal positional encoding is added, and a 2-layer Transformer encoder (consisting of four attention heads and a feedforward dimension of 256) processes the sequence. The final time-step representation is used for classification. This model is also trained with Adam (learning rate of 0.001) over 20 epochs, latency and throughput are reported in Table 2.

#### 3.3.3. Hybrid Models

The hybrid models combine CNN-based feature extraction with additional sequential models or classical classifiers. This design leverages local feature extraction (via convolutional layers) and augments it with temporal or ensemble modeling, enhancing performance under both single-device and cross-device scenarios. To ensure reproducibility and clarity, we provide detailed pseudocode for each hybrid architecture in Algorithms 1–3.

- Hybrid CNN + LSTM: Each normalized signal is reshaped to (75,100) and passed through a CNN block consisting of a 1D convolution (kernel size of 3 with 16 filters), batch normalization, ReLU activation, and max pooling (kernel size of 2), resulting in a feature map of approximately (B,37,16), where *B* is the batch size. An LSTM then processes this feature map with a hidden size of 128. The final hidden state is classified via a fully connected layer. Training is conducted over 20 epochs with Adam, where learning rate is 0.001, and achieves 97–98% accuracy with a throughput up to 161K samples/s. Algorithm 1 describes the Hybrid CNN + LSTM model development process.
- Hybrid CNN + Transformer: After normalization and reshaping to (75,100), the signal is processed by the same CNN block as in the CNN + LSTM model to yield a feature map of shape (B,L,16), with L≈37. This feature map is linearly projected to the transformer dimension, augmented with positional encodings, and processed by a 2-layer Transformer encoder (with four heads and a feedforward dimension of 256). The final time-step representation is then classified via a fully connected layer. Training is performed for 20 epochs (Adam, learning rate = 0.001) with comparable accuracy to the CNN + LSTM hybrid. Algorithm 2 describes the Hybrid CNN + Transformer pipeline.
- Hybrid CNN + RF: For this approach, each raw signal (reshaped to (1,7500)) is normalized and passed through a CNN block (conv, BN, ReLU, max pooling) to extract a feature embedding (e.g., 128-dimensional). The embeddings are then used as inputs to a Random Forest classifier. Embeddings are extracted using a CNN and classified using a pre-trained RF during inference. The CNN is trained normally, and then embeddings are extracted and used to train an RF. This yields about 95% accuracy on single-device data.

**Algorithm 1:** Hybrid CNN + LSTM Model

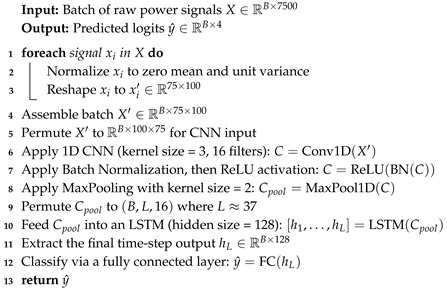



**Algorithm 2:** Hybrid CNN + Transformer Model

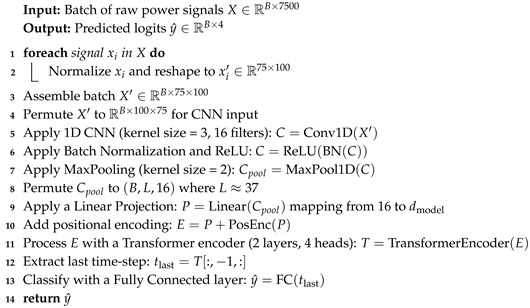



**Algorithm 3:** Hybrid CNN + RF Model

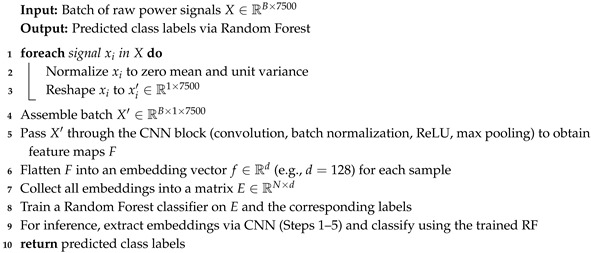



Each hybrid model specification details how raw power consumption data is processed through a CNN and then further modeled by an LSTM, Transformer, or RF classifier. These subsections clarify the sequential operations and provide a foundation for discussing computational efficiency and overall performance.

## 4. Performance Evaluation

This section presents the evaluation results of our selected learning models on different scenarios described in Section 3. First, we present the evaluation methodology, then the evaluation results, and finally provide analysis, observations, and insights.

### 4.1. Evaluation Methodology

To thoroughly assess the performance and generalization capability of our models, we conducted a series of experiments using both single-device and cross-device configurations on the CHASE’19 dataset. To ensure the statistical robustness of our findings and mitigate the risks of overfitting or partitioning bias, we adopted a rigorous dual-validation strategy. First, we employed 5-fold cross-validation for all experiments to establish stable mean performance metrics (reported as Mean ± SD). Second, we executed three independent training runs with different random seeds (42, 43, 44) to identify the best-performing model configuration and quantify sensitivity to initialization. This approach confirms that the high accuracies reported are reproducible and not artifacts of a specific data split.

In addition to classification performance, we report computational efficiency as average inference time per batch (s/batch) and throughput (samples/s). These metrics help assess the practicality of each model in real-time or resource-constrained IoT environments.

To validate the robustness of our models, we further evaluated their performance on the Three-Class Botnet Dataset [30]. For this evaluation, we utilized the balanced version (3000 samples per class) to test the models’ ability to learn from limited data without the confounding factor of class imbalance. This evaluation provides insight into the models’ adaptability in scenarios where balanced and abundant data is not guaranteed, reflecting realistic deployment conditions in IoT systems. Through the section, the router dataset refers to the CHASE’19 dataset, which contains data collected exclusively from router devices. The combined dataset, on the other hand, includes CHASE’19 data gathered from all three device types: routers, cameras, and voice assistants.

In the following, we present our evaluation results, starting with single-device experiments, proceeding to cross-device evaluations, and then evaluating the Three-Class Botnet Dataset [30]. The analysis concludes with a comparative assessment of computational efficiency and a leave-one-device-out evaluation.

### 4.2. Evaluation Results

#### 4.2.1. Single-Device Experiments

We trained and evaluated models independently on CHASE’19 datasets for each IoT device (Router, Camera, and Voice Assistant), and Table 2 summarizes classification performance and efficiency metrics for classical, deep, and hybrid models. As detailed in Section 4.3, hybrid models, especially CNN + Transformer and CNN + RF, consistently offer the best balance between accuracy and computational efficiency, while classical models like SVM lag in both accuracy and generalization.

To supplement our performance assessment, we evaluated results on the balanced three-class botnet dataset (9000 total samples). We performed a strict leakage check (verifying 0% overlap between train/test splits). As shown in Table 3, the models achieve near-perfect accuracy (100%) across all three random seeds (42, 43, 44). This confirms that the power signatures of infected devices are highly distinguishable from idle and benign states.

##### CNN Model Performance

**Figure 3:**
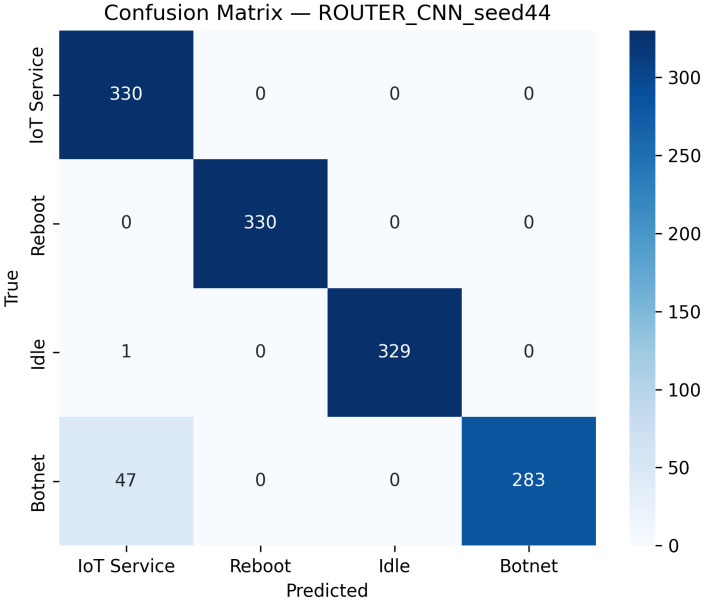
Confusion matrix of the CNN model evaluated on the Router dataset using the best seed (Seed 44). The model demonstrates excellent accuracy and balanced classification performance across all traffic categories.

**Figure 4:**
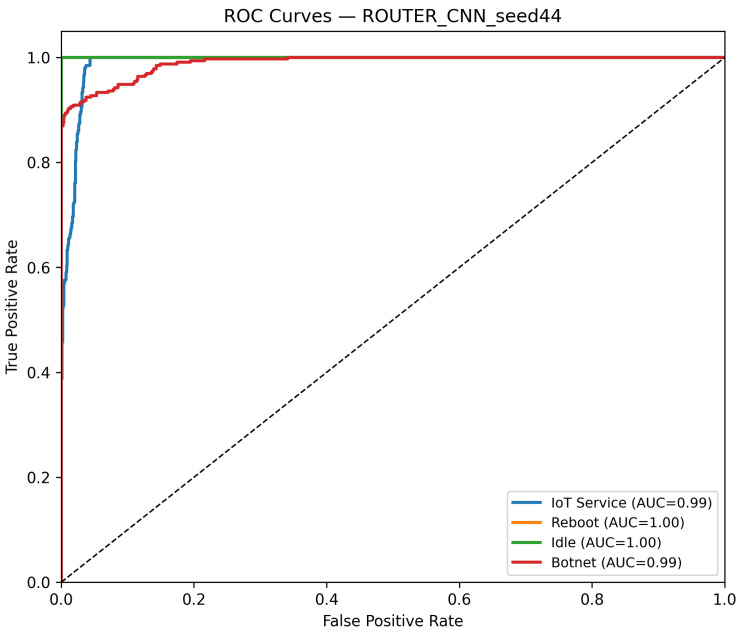
ROC curves of the CNN model on the Router dataset (Seed 44). The diagonal dashed line denotes the random-chance baseline. The Reboot (orange) curve overlaps with other classes due to identical performance (AUC = 1.00).

The CNN model is evaluated on all the distinct device datasets, including routers, cameras, and voice assistants. While Table 2 presents overall performance, Table 4 specifically summarizes class-specific precision, recall, and F1-score on the router dataset. On the router device, the best-seed configuration (Seed 44) achieves 96.36% test accuracy and 96.35% macro-F1, with an average inference time of 0.00046 s per batch and an approximate throughput of 87,485 samples per second. Figure 3 shows the confusion matrix. Additionally, Figure 4 displays the ROC curves for this model.

##### LSTM Model Performance

**Figure 5:**
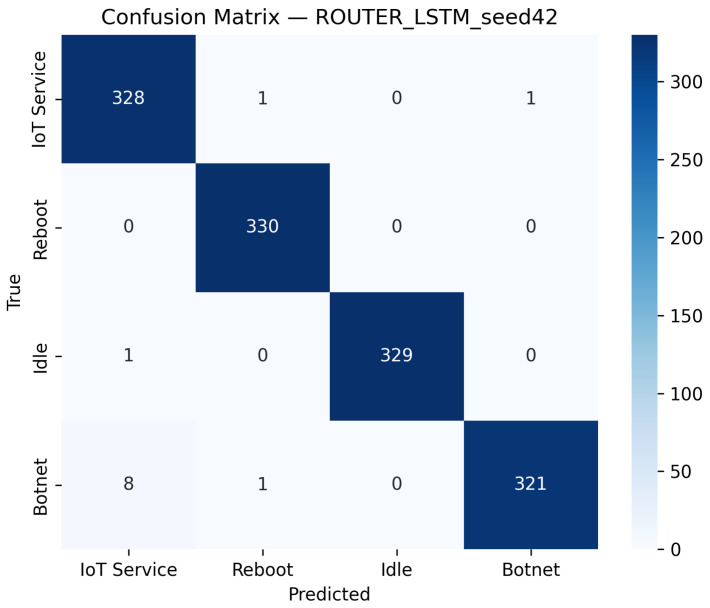
Confusion matrix of the LSTM model on the Router dataset using the best-performing seed (Seed 42).

**Figure 6:**
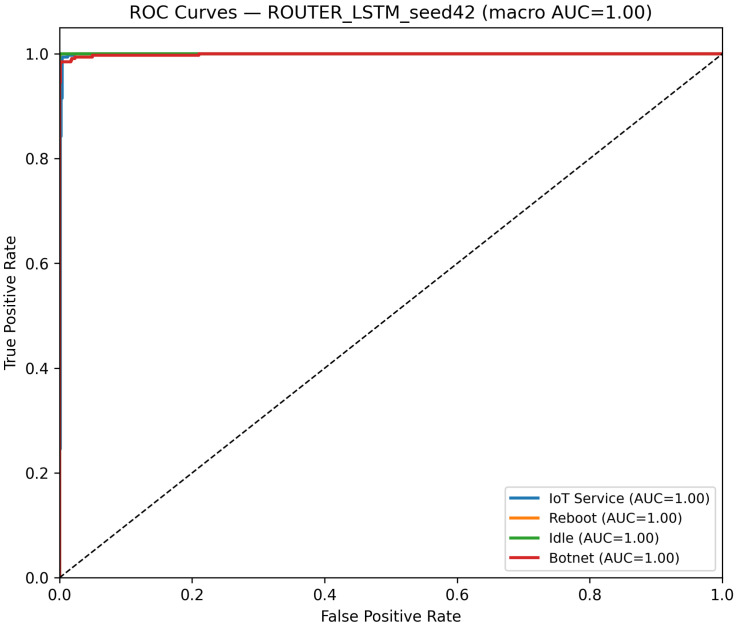
ROC curves of the LSTM model on the Router dataset (Seed 42). The diagonal dashed line denotes the random-chance baseline. The Reboot (orange) curve overlaps with other classes due to identical performance (AUC = 1.00).

The LSTM model, which processes input signals reshaped to (75,100), is evaluated in a similar manner. Its performance on all distinct devices is reported in Table 2, with Table 5 presenting its metrics performance on Router Devices. As shown in Figure 5 and Figure 6, the LSTM model achieves a test accuracy of 99.09% and a macro-F1 of 99.09% on the router dataset for the best seed (Seed 42), while maintaining an average inference time of 0.00060 s per batch and throughput of roughly 105,000 samples per second (Table 2). The per-class precision, recall, and F1 scores indicate robust performance across all classes.

The confusion matrix, as shown in Figure 5, illustrates that misclassifications are minimal, while the ROC curves, as shown in Figure 6, confirm strong discriminative performance with high AUC values. Overall, the LSTM model’s performance is comparable to that of the CNN model, effectively leveraging sequential information to achieve high classification accuracy.

##### 1D Transformer Model Performance

The 1D Transformer processes signals reshaped to (75,100) using a linear embedding and sinusoidal positional encoding. For this model, the test accuracy on the router dataset is 97.80% with a macro-F1 of 97.80% for the best-performing seed (Seed 42), and an average inference time of 0.00080 s per batch, corresponding to a throughput of approximately 64,709 samples per second on the last batch (Table 2 and Table 6).

Table 6 summarizes the detailed classification performance metrics for each class evaluated on the router dataset. Table 2 presents the performance on all distinct devices.

**Figure 7:**
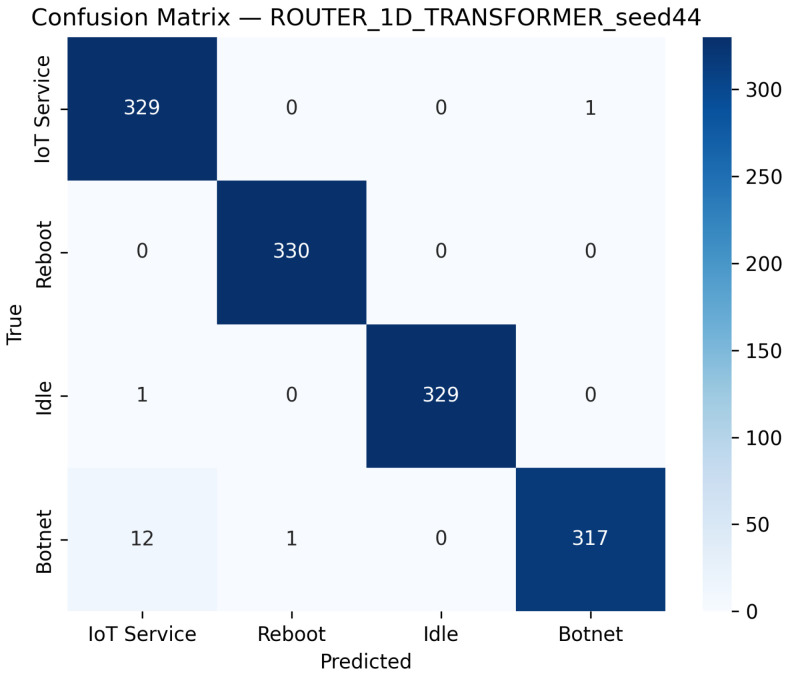
Confusion matrix of the 1D Transformer model on the Router dataset using the best seed (Seed 44).

**Figure 8:**
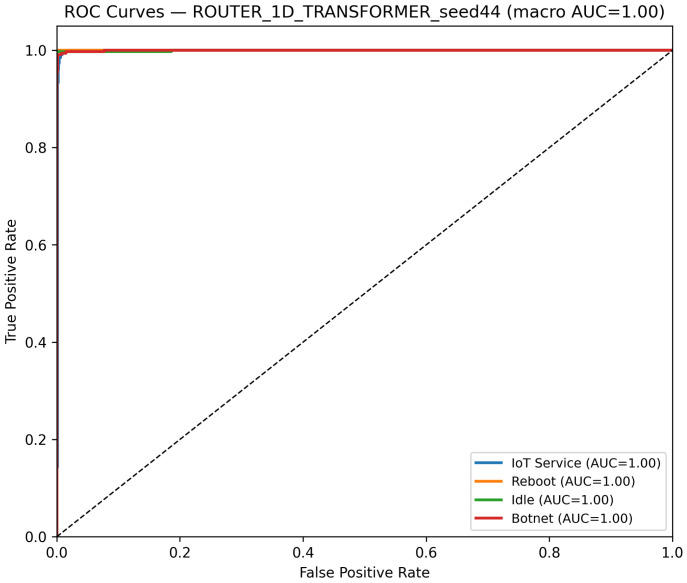
ROC curves for the 1D Transformer model on the router dataset best seed (Seed 44). The dashed diagonal indicates the random-chance baseline. Class curves overlap due to identical performance (AUC = 1.00).

Additionally, Figure 7 illustrates the confusion matrix for the 1D Transformer model on the router dataset, indicating that the model achieves robust classification with only minor misclassifications. Similarly, Figure 8 presents the ROC curves for each class, confirming high AUC values and strong discriminative performance.

These figures collectively demonstrate that, although the 1D Transformer performs slightly worse than the CNN and LSTM models, it still delivers robust classification results with high overall accuracy and excellent AUC values.

#### 4.2.2. Cross-Device Experiments

The cross-device experiments evaluate model performance on a combined device dataset that aggregates data from all device types (routers, cameras, and voice assistants). This combined device dataset (26,400 windows × 7500 samples) enables us to assess the generalization capabilities of each model across heterogeneous devices. In the following subsections, we detail the performance of each approach on the cross-device data, highlighting classification metrics, confusion matrices, and ROC curves.

##### Classical Models on Combined Data

For classical methods, the engineered 11-dimensional feature vectors are extracted from the combined signals. Table 7 compares the classification performance of SVM and RF on the combined device dataset. In our experiments, Random Forest achieves nearly 100% accuracy, whereas SVM performance is significantly lower (around 78%).

##### Deep Models on Combined Data

For the CNN, LSTM, and 1D Transformer models applied to the combined device dataset, Table 7 summarizes the overall performance (accuracy, precision, recall, and F1-score). Additionally, confusion matrices and ROC curves for each model offer insight into the performance of each class.

**Figure 9:**
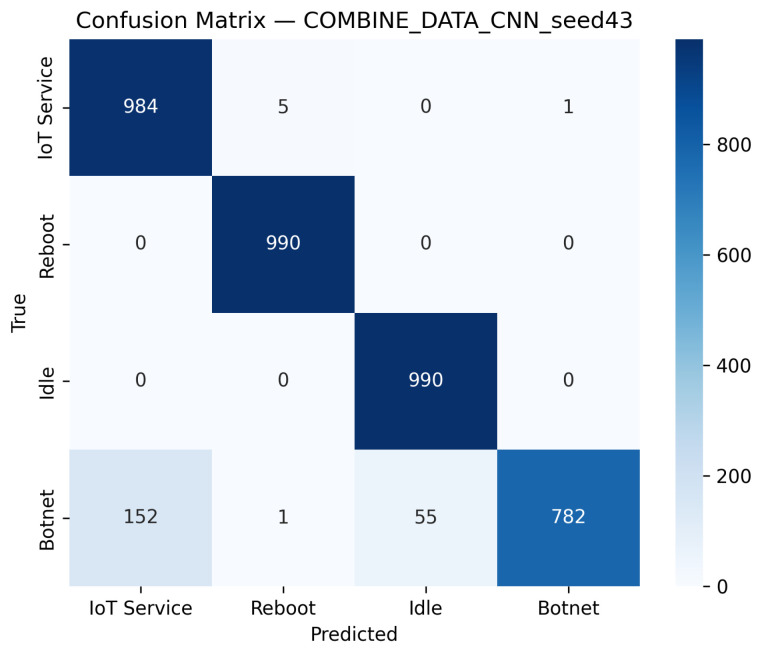
Confusion matrix for the CNN model on the combined device data, best seed (43).

**Figure 10:**
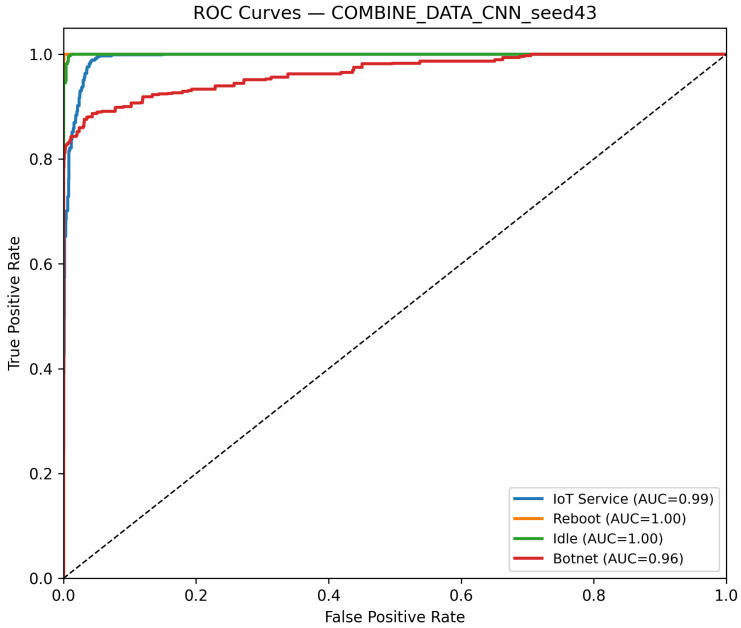
ROC curves for the CNN model on the combined device data, best seed (43). The diagonal dashed line denotes the random-chance baseline. The Reboot (orange) curve overlaps with other classes due to identical performance (AUC = 1.00).

**Figure 11:**
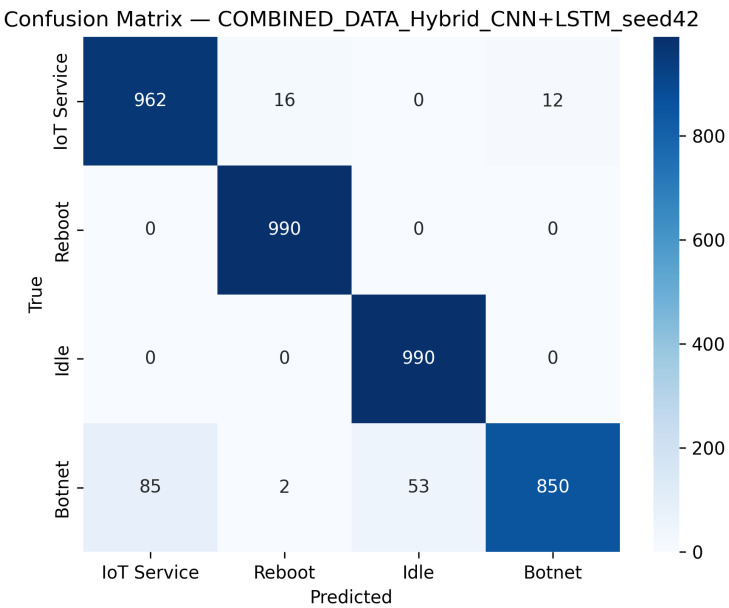
Confusion matrix of the CNN + LSTM hybrid model on the combined multi-device dataset using the best seed (Seed 42).

**Figure 12:**
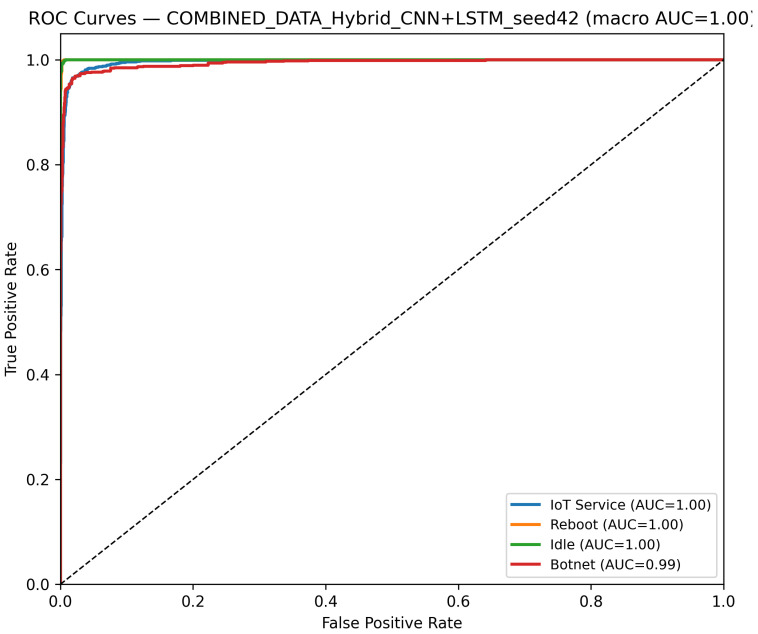
ROC curves of the CNN+LSTM hybrid model on the combined dataset (Seed 42). The diagonal dashed line denotes the random-chance baseline. The Reboot (orange) curve overlaps with other classes due to identical performance (AUC = 1.00).

**Figure 13:**
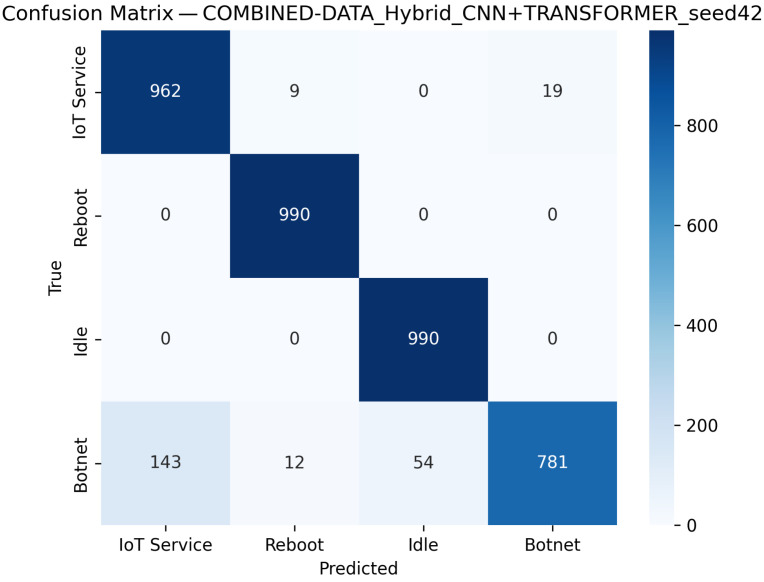
Confusion matrix of the CNN+Transformer model on the combined dataset using the best-performing seed (Seed 42).

**Figure 14:**
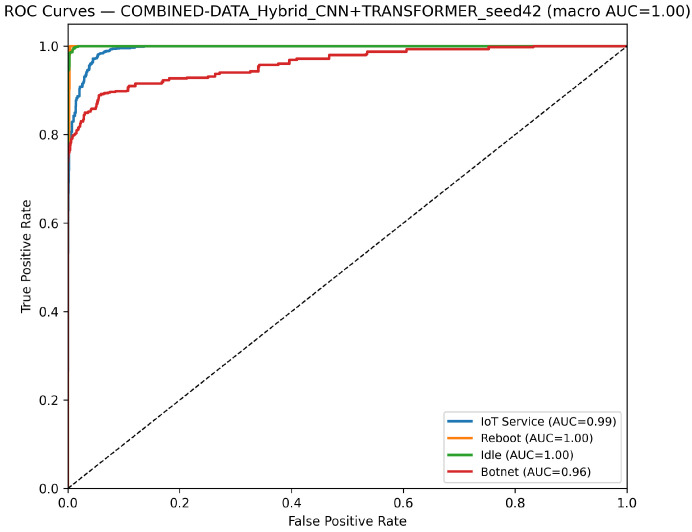
ROC curves for Hybrid CNN + Transformer model on combined device dataset (Seed 42). The dashed diagonal indicates the random-chance baseline. Class curves overlap due to identical performance (AUC = 1.00).

**Figure 15:**
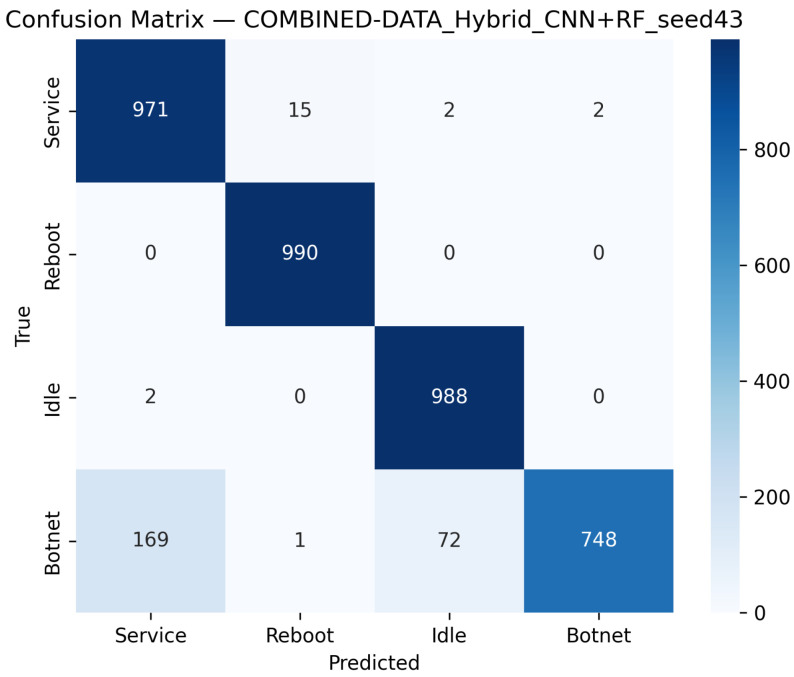
Confusion matrix of the CNN+RF model on the combined dataset using the best seed (Seed 43).

**Figure 16:**
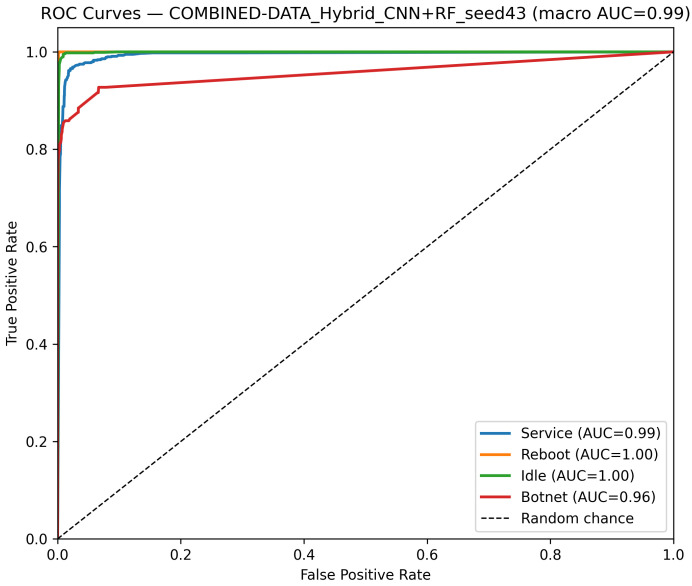
ROC curves of the CNN + RF model on the combined dataset (Seed 43). The dashed diagonal indicates the random-chance baseline. Class curves overlap due to identical performance (AUC = 1.00).

- CNN Model (Combined Device Data): Figure 9 illustrates the confusion matrix for the CNN model when applied to the combined device dataset. The matrix indicates that the model maintains high true positive rates across most classes, although slight performance degradations can be observed compared to single-device experiments. Figure 10 illustrates the ROC curves with strong AUC values for each class.
  For the LSTM and 1D Transformer models, similar evaluations (with corresponding tables and discussion) are performed on the combined device dataset.
- Hybrid Models on Combined Data: Hybrid models, which integrate CNN-based feature extraction with sequential modules or classical classifiers, are critical for achieving robust cross-device performance. Their evaluations on the combined device dataset yield insights into both classification performance and computational efficiency.
- Hybrid CNN + LSTM (Combined Device Data): Figure 11 presents the confusion matrix for the CNN + LSTM model on the combined device dataset, while Figure 12 shows its ROC curves. These figures indicate that although the hybrid approach slightly reduces overall accuracy compared to single-device experiments, it still retains strong per-class performance.
- Hybrid CNN + Transformer (Combined Device Data): For the CNN + Transformer model, Figure 13 and Figure 14 display the confusion matrix and ROC curves, respectively. The results confirm that the model leads to robust performance when evaluated across heterogeneous data.
- Hybrid CNN + RF (Combined Device Data): The Hybrid CNN + RF model is evaluated by extracting CNN-based embeddings from the combined device dataset and classifying them using a Random Forest. Its performance is summarized in Table 7 and visualized in Figure 15 and Figure 16.

#### 4.2.3. Computational Efficiency Comparison

Table 8 presents the inference efficiency measured in average inference time and throughput (samples/s).

### 4.3. Performance Analysis

This section discusses and analyzes the performance of classical, deep, and hybrid machine learning models across two evaluation settings: (i) single-device, (ii) cross-device, and (iii) leave-one-device-out. Each experiment assesses classification accuracy, computational efficiency (inference time, throughput, training time), and generalization across IoT devices. The section concludes by identifying the most balanced model concerning predictive performance and real-time suitability.

#### 4.3.1. Single-Device Evaluation

##### Model Performance Comparison

In the single-device setting, the models were trained and tested separately on data from three IoT devices: Router, Camera, and Voice Assistant. Classical learning models displayed divergent outcomes, as presented in Table 2. SVM consistently underperformed, with accuracies ranging from 27.05% (Router and Camera) to 39.94% (Voice Assistant), highlighting limitations in capturing nonlinear dynamics and in handling class imbalance. In contrast, RF achieved outstanding performance across all devices, with accuracies of 99.03% (Router), 98.81% (Camera), and 99.43% (Voice Assistant) and macro-F1 values that match test accuracy, since the classes are well balanced, demonstrating its robustness even with limited statistical features.

Deep learning models also achieved strong single-device performance. For CNNs, the best-seed test accuracies span 92.42–96.36% across devices (Voice Assistant, Camera, and Router), with macro-F1 closely tracking accuracy (Table 2). LSTM networks, designed for temporal sequence modeling, yield best-seed test accuracies of 99.09%, 95.53%, and 88.71% on the Router, Camera, and Voice Assistant devices, respectively, again with macro-F1 nearly identical to accuracy. The 1D Transformer model provides competitive performance, with best-seed test accuracies of 98.86% (Router, Seed 44), 95.83% (Camera, Seed 42), and 86.36% (Voice Assistant, Seed 42) and macro-F1 values within 1% of these accuracies. Table 2 summarizes these deep learning results for all three IoT devices.

Hybrid architectures emerge as the most flexible single-device models. Table 2 shows that CNN + LSTM achieves best-seed test accuracies between 91.74% and 95.15% with macro-F1 values that track accuracy, effectively capturing both spatial and temporal features. CNN + Transformer surpasses other deep and hybrid models on several devices, with peak single-device accuracy of 99.39% (Router) and macro-F1 of 99.40%, while CNN + RF, which uses CNN embeddings fed into an RF classifier, attains best-seed accuracies of 96.52%, 91.97%, and 86.67% across Router, Camera, and Voice Assistant, respectively.

##### Efficiency and Accuracy Trade-Offs

From a computational standpoint, classical models like SVM offered fast inference (<0.001 s/batch) but at the cost of low accuracy. RF models were accurate but had longer inference times 0.00635–0.00918 s/batch across devices. Deep learning models strike a balance between accuracy and efficiency. CNNs demonstrate low inference latency (∼0.00058 s/batch) and high throughput, with best-seed throughputs consistently around $1.0\times 10^{5}$ samples/s across all devices as summarized in Table 2. LSTM networks had slightly higher inference time (about 5.9–$6.0\times 10^{-4}$ s/batch) but longer training durations (>6 s). The 1D Transformer model yielded a solid performance with inference times around $8\times 10^{-4}$ s/batch and throughput between $3.7\times 10^{4}$ and $6.5\times 10^{4}$ samples/s, depending on the device.

Hybrid models optimized both performance and speed. CNN + LSTM achieved throughputs on the order of 7–$8\times 10^{4}$ samples/s and accuracies between about 92% and 95% across devices. CNN + Transformer achieved up to 99.39% accuracy with a throughput of roughly 6.0–$6.3\times 10^{4}$ samples per second. CNN + RF was remarkably efficient, delivering between $1.1\times 10^{5}$ (Router) and $1.3\times 10^{5}$ (Voice Assistant) samples/s, marking it as a strong candidate for real-time edge deployments. On average, across all three devices, CNN + Transformer and CNN + LSTM offered the best performance balance among hybrid models.

#### 4.3.2. Cross-Device Evaluation

##### Model Performance Comparison

The cross-device experiments evaluated models on a combined device dataset representing all devices, reflecting a more realistic and challenging deployment scenario. Among classical models, SVM achieved 75.00% accuracy, again showing its limited generalizability compared to the best deep and hybrid models. RF continued to demonstrate exceptional robustness, reaching 99.44% accuracy with high macro-F1 (99.44%) on the combined dataset (Table 7).

Deep learning models also retain strong cross-device performance. On the combined dataset, the best-seed test accuracies for CNN, LSTM, and the 1D Transformer are 94.60% (Seed 43), 94.42% (Seed 44), and 93.16% (Seed 44), respectively, with macro-F1 values that closely match test accuracy as presented in Table 7. These results validate the capacity of deep models to generalize across diverse device behaviors, particularly those that incorporate temporal dynamics.

Hybrid models slightly outperform the deep baselines in this cross-device setting. CNN + LSTM attains 95.76% test accuracy (Seed 42) and 94.13% macro-F1, while CNN + RF reaches 93.36% accuracy and 93.14% macro-F1. CNN + Transformer emerges as a strong cross-device candidate, with a best-seed test accuracy of 94.02% (Seed 42) and macro-F1 of 97.81% on the combined dataset, as summarized in Table 7.

##### Efficiency and Accuracy Trade-Offs

In terms of inference latency and throughput, the CNN + RF hybrid achieved the highest throughput of approximately $1.67\times 10^{5}$ samples/s in the cross-device setting with an average inference time of 0.02323 s/batch, suggesting suitability for high-throughput classification when a CNN feature extractor can run ahead of the RF classifier. CNN + Transformer maintained competitive throughput about $7.0\times 10^{4}$ samples/s with an average inference time of 0.00075 s/batch with excellent accuracy. CNN + LSTM offered high throughput approximately $1.1\times 10^{5}$ samples/s at 0.00053 s/batch and strong accuracy, presenting a reliable compromise between the advantages of CNN and Transformer.

Notably, while RF maintained top accuracy among classical models, its inference time (0.00815 s/batch) limits its utility in latency-sensitive scenarios on constrained hardware. SVM remains fast at around 0.00147 s/batch, but continues to show poor generalization, with single-device accuracies of 27.05–39.94% and a cross-device accuracy of only 78.26%, as Table 7 summarizes, making it unsuitable for deployment compared to modern deep and hybrid models. Deep models, such as LSTM and Transformer, maintained robust throughput and inference profiles, albeit with relatively higher training times than CNNs.

#### 4.3.3. LODO Evaluation Results

In this experiment, we trained the models using data from two devices in the CHASE’19 dataset and test their ability to diagnose data from the third, previously unseen, device. Table 9 summarizes the Leave-One-Device-Out (LODO) evaluation, where the Voice Assistant device was excluded during training. The table compares classical, deep, and hybrid models in terms of accuracy, precision, recall, F1-score, inference latency, throughput, and training time. The LODO test is a critical evaluation strategy for assessing the generalization capability of our models in device-heterogeneous environments. Our results demonstrate that our model effectively adapts to variations in device characteristics, configurations, and behaviors without the need for retraining.

#### 4.3.4. Best Model Trade-Off Analysis

Considering both single- and cross-device evaluations, hybrid models lead to the best trade-off between classification performance and computational efficiency. The CNN + Transformer model is the most accurate across both settings, demonstrating superior generalization, especially under heterogeneous conditions. However, for deployments prioritizing speed and scalability, such as edge-based real-time intrusion detection, CNN + RF provides the optimal balance, offering competitive accuracy (∼96%) and the highest throughput (>153K samples/s).

In summary, hybrid models benefit from the complementary strengths of CNN-based feature extraction and either sequential modeling (LSTM or Transformer) or classical classification (RF). Among them, CNN + Transformer is best suited for accuracy-driven deployments, while CNN + RF excels in real-time, resource-efficient systems.

## 5. Limitations and Threats to Validity

This study is positioned as a unified benchmarking exercise. The evidence we present is therefore bounded by the datasets, splits, and measurement protocol we adopted.

- While the CHASE’19 corpus enables consistent single-device, cross-device, and leave-one-device-out evaluations within a single source, it reflects devices and attack mixes that are now older. As a result, headline numbers may not fully anticipate changes in firmware, power management, or newer botnet families. We use CHASE’19 to preserve comparability with prior power-based IDS work and to isolate modeling and deployment trade-offs; however, we acknowledge that broader external validation remains necessary.
- Device diversity in our experiments is limited to three categories: routers, cameras, and voice assistants. This narrows the space of hardware states, idle behaviors, and power delivery conditions. Cross-device and leave-one-device-out protocols reduce partition bias, but they do not substitute for systematic evaluations on additional device classes, different power monitors, or mains conditions that vary across environments.
- All models were trained and evaluated on fixed 1.5 s windows with balanced splits for CHASE’19. Fixed windows aid reproducibility and controlled comparison, yet they may smooth longer temporal dynamics and reduce sensitivity to bursty or slowly evolving behaviors. Class balancing simplifies learning in homogeneous settings, although it can move the operating point away from natural prevalence in deployment.
- Regarding the Three-Class Botnet Dataset used for robustness checks, our rigorous validation (using 5-fold cross-validation and multiple random seeds) consistently yielded 100% accuracy across all folds. Despite ensuring strict separation between training and testing sets to prevent data leakage, this uniform perfection suggests that the specific attack signatures in this smaller dataset (e.g., high-intensity DDoS power spikes) are highly separable from the idle/benign baselines. Consequently, while these results confirm the models’ ability to distinguish distinct anomalies, this dataset may lack the complexity or noise required to stress-test deep architectures or differentiate fine-grained model performance compared to the CHASE’19 benchmark.
- Our efficiency measurements report average inference latency and throughput under a single hardware and software stack, including warm-up. These measurements capture relative trends between model families, however absolute values can change with batch size, kernel implementations, scheduler variability, and different accelerators. Energy per inference and end-to-end system latency on embedded platforms were not measured in this study.
- Finally, this work does not evaluate zero-day families or adversaries that attempt to obscure or perturb power signatures. We also do not include interpretability analyses beyond aggregate metrics. Both aspects are important for practical adoption and for diagnosing failure modes under device shift.

## 6. Extension

Building on the unified benchmark and the results in Table 2, Table 7 and Table 9, we outline three complementary directions that follow naturally from the observed accuracy and efficiency trade-offs and from the cross-device and LODO findings to extend the current pipeline.

- Future Data Acquisition and Evaluation: We plan to extend the benchmark with newer devices and firmware, a wider set of benign scenarios, and additional botnet families. We will expand cross-dataset testing, include calibrated precision–recall at fixed false alarm rates, and report energy per inference and on-device latency. We also intend to add repeated trials with multiple seeds, training curves, and per-class operating points, then release matched splits and scripts to enable apples-to-apples comparisons on future corpora.
- Larger-scale benchmarking across datasets and models: In this direction, we will expand the unified benchmark by adding more public and in-house datasets and by including additional model families. On the model side, this includes temporal convolutional variants, lightweight 1D architectures suitable for embedded use, and gradient-boosted trees trained on learned embeddings. On the data side, we will diversify device types and operating conditions to stress-test single-device, cross-device, and leave-one-device-out protocols under broader distribution shift. All preprocessing, training, and evaluation scripts will follow the same standardized pipeline used in this paper to ensure reproducibility.
- Cross-device adaptation and calibration-free transfer: To improve robustness under device shift, we will explore source-only domain generalization, test-time adaptation using batch statistics, and few-shot calibration that uses short unlabeled power windows collected on a new device. These methods will be assessed using the same metrics and protocols as before, with additional stress tests for firmware updates, sampling rate changes, and operating mode drift.
- Edge co-design with efficiency profiling and compression: To translate the best models into deployable systems, we will combine quantization, pruning, and knowledge distillation with the latency and throughput measurements already reported. We will extend efficiency profiling to include energy per inference and end-to-end latency on representative gateways and microcontrollers, and then report accuracy, latency, throughput, and energy together to guide practical deployments.
- Explainable AI (XAI) integration: To address the “black box” nature of deep learning models and enhance trust in edge deployment, we will integrate post-hoc interpretability frameworks such as SHAP (SHapley Additive exPlanations) and LIME (Local Interpretable Model-agnostic Explanations). This will allow us to map specific power consumption features (e.g., CPU frequency spikes vs. transceiver idle states) to model predictions, providing security analysts with actionable insights beyond raw classification scores.

These directions integrate directly with our existing workflow, enabling a broader and more deployment-focused evaluation in future work.

## 7. Final Remarks

In this paper, we have evaluated a broad spectrum of models, ranging from classical (SVM and RF) to deep (CNN, LSTM, and 1D Transformer) to hybrid architectures (CNN + LSTM, CNN + Transformer, and CNN + RF) using the CHASE’19 dataset. Our experiments demonstrate that while deep models achieve high accuracy in homogeneous settings, their performance often deteriorates under cross-device conditions. Classical models, such as Random Forest, consistently deliver robust generalization, while hybrid models strike a practical balance between accuracy and efficiency.

Furthermore, our computational efficiency analysis shows that many of these models, particularly the hybrid ones, are well-suited for real-time deployment on edge devices due to their low inference latency and high throughput. Additionally, evaluation on the Three-Class Botnet Dataset has confirmed the robustness of our models—particularly the CNN and hybrid architectures—even in scenarios with highly imbalanced data and a limited number of samples. This work presents a comprehensive evaluation of classical, deep, and hybrid machine learning architectures for power-based IoT botnet detection, highlighting key trade-offs in generalization and computational efficiency across heterogeneous devices.

## Figures and Tables

**Table 1:** Key hyperparameters used across models.

Model Family	Specific Hyperparameters
Classical	Random Forest: Trees = 100, Max Depth = 20, Criterion = Gini
	SVM: Kernel = RBF, C=10, γ=auto
Deep Learning	CNN: Conv1D(10 filters, k = 512, s = 128), BN, ReLU, MaxPool(4), FC
	LSTM: Input = (75, 100), Hidden = 128, Layers = 1, Dropout = 0.0
	Transformer: Layers = 2, Heads = 4, $d_{model}=128$, $d_{ff}=256$, Sinusoidal PE
Hybrid	CNN + LSTM: CNN block (as above) + LSTM (Hidden = 128)
	CNN + Transformer: CNN block (as above) + Transformer (as above)
	CNN + RF: CNN Feature Extractor + RF Classifier (as above)
Training Config	Optimizer = Adam, Learning Rate = $1\times 10^{-3}$, Epochs = 20

**Table 2:** Performance and efficiency of all models on single-device datasets (best seed results).

Model (Device)	Test Acc. (Best Seed)	Macro-F1 (Best Seed)	K-Fold Acc. (Mean ± Std)	Avg. Inf. Time (s/Batch)	Throughput (Samples/s)
Classical Models					
SVM (Router)	27.05%	13.95%	26.93±0.35%	0.63533	2770
SVM (Camera)	27.05%	13.95%	26.93±0.35%	0.63486	2773
SVM (Voice Assistant)	40.74%	32.71%	40.36±0.83%	0.55116	3194
SVM (Router)	27.05%	13.95%	26.99±0.31%	0.6355	2769
SVM (Camera)	27.05%	13.95%	26.99±0.31%	0.6258	2812
SVM (Voice Assistant)	40.74%	32.71%	40.45±0.38%	0.5443	3233
Deep Learning					
CNN (Router)	96.36%	96.35%	98.60±0.34%	0.00058	108,840
CNN (Camera)	93.94%	93.94%	96.41±0.49%	0.00058	109,408
CNN (Voice Assistant)	92.42%	92.27%	98.75±0.45%	0.00058	109,321
LSTM (Router)	99.09%	99.09%	99.15±0.11%	0.00060	104,903
LSTM (Camera)	95.53%	95.51%	98.65±0.17%	0.00059	105,707
LSTM (Voice Assistant)	88.71%	87.77%	99.28±0.26%	0.00059	106,387
1D Transformer (Router)	98.86%	98.86%	98.24±0.24%	0.00119	52,864
1D Transformer (Camera)	95.83%	95.83%	96.35±0.14%	0.00123	51,100
1D Transformer (Voice Assistant)	86.36%	84.88%	98.31±0.52%	0.00119	52,695
Hybrid Learning					
CNN + LSTM (Router)	95.15%	95.10%	99.16±0.12%	0.00069	92,566
CNN + LSTM (Camera)	93.33%	93.22%	96.59±0.46%	0.00068	93,439
CNN + LSTM (Voice Assistant)	91.74%	91.55%	99.55±0.14%	0.00065	96,781
CNN + Transformer (Router)	99.39%	99.40%	99.02±0.19%	0.00092	68,235
CNN + Transformer (Camera)	96.36%	96.36%	97.30±0.26%	0.00109	57,629
CNN + Transformer (Voice Assistant)	97.12%	97.11%	99.66±0.09%	0.00103	61,019
CNN + RF (Router)	96.52%	96.49%	96.25±0.74%	0.0117	113,535
CNN + RF (Camera)	91.97%	91.64%	90.14±0.45%	0.0108	163,000
CNN + RF (Voice Assistant)	86.67%	85.68%	96.57±0.23%	0.0094	188,000

**Table 3:** Classification Performance on Three-Class Botnet Dataset.

Model	Accuracy (%)	Precision	Recall	F1-Score
CNN	100.00	1.00	1.00	1.00
LSTM	100.00	1.00	1.00	1.00
1D Transformer	100.00	1.00	1.00	1.00
Random Forest	100.00	1.00	1.00	1.00
SVM	84.00	0.88	0.84	0.83
CNN + LSTM	100.00	1.00	1.00	1.00
CNN + Transformer	100.00	1.00	1.00	1.00
CNN + RF	100.00	1.00	1.00	1.00

**Table 4:** CNN Model Performance on the Router Dataset (Best Seed = 44).

Class	Precision	Recall	F1-Score
IoT Service	0.8730	1.0000	0.9322
Reboot	1.0000	1.0000	1.0000
Idle	1.0000	0.9970	0.9985
Botnet	1.0000	0.8576	0.9233
Overall Accuracy	96.36%		

**Table 5:** LSTM Model Performance on the Router Dataset (best seed).

Class	Precision	Recall	F1-Score
IoT Service	0.97	0.99	0.98
Reboot	0.99	1.00	1.00
Idle	1.00	1.00	1.00
Botnet	1.00	0.97	0.98
Overall Accuracy	99.09%		

**Table 6:** One-Dimensional Transformer Model Performance on the Router Dataset (best seed).

Class	Precision	Recall	F1-Score	Support
IoT Service	0.96	0.99	0.97	324
Reboot	1.00	1.00	1.00	334
Idle	1.00	0.99	1.00	333
Botnet	1.00	0.95	0.97	329
Overall Accuracy	97.80%			

**Table 7:** Classification performance and efficiency on the cross-device (combined) dataset using best-seed test results and k-fold validation statistics.

Model	Test Acc. (Best Seed)	Macro-F1 (Best Seed)	K-Fold Acc. (Mean ± Std)	Avg. Inf. Time (s/Batch)	Throughput (Samples/s)
RF	99.58%	99.58%	99.49±0.09%	0.00808	59,566
SVM	79.07%	76.20%	78.23±0.34%	3.73637	1413
CNN	94.60%	94.44%	97.39±0.17%	0.00054	118,688
LSTM	94.42%	94.31%	98.95±0.13%	0.00060	107,023
1D Transformer	93.16%	93.03%	97.49±0.34%	0.00119	53,898
CNN + LSTM	94.12%	94.13%	97.54±0.19%	0.00053	114,025
CNN + Transformer	94.02%	93.85%	98.29±0.12%	0.00119	53,682
CNN + RF	93.36%	93.14%	95.31±0.26%	0.0238	166,726

**Table 8:** Computational efficiency on the cross-device dataset using best-seed repeated-seed runs.

Model	Avg. Inference Time (s/Batch)	Throughput (Samples/s)
RF	0.00808	59,566±3597
SVM	3.73637	1413±18
CNN	0.000541	118,688±10,490
LSTM	0.000600	107,023±9037
1D Transformer	0.00119	53,898±658
CNN + LSTM	0.000573	121,345±7214
CNN + Transformer	0.001067	60,338±6746
CNN + RF	0.0238	166,726±4482

**Table 9:** LODO Evaluation Results (Voice Assistant Left Out During Training).

Model	Accuracy (%)	Precision	Recall	F1-Score	Avg. Inference Time (s/Batch)	Throughput (Samples/s)	Training Time (s)
Classical Models							
SVM	75.00	0.62	0.75	0.67	0.0015199	–	N/A
RF	62.95	0.71	0.63	0.56	0.0077908	–	N/A
Deep Learning Models							
CNN	74.91	0.78	0.75	0.73	0.000382	83,822	17.00
LSTM	78.52	0.83	0.79	0.77	0.0001677	–	14.43
1D Transformer	77.84	0.81	0.78	0.77	0.0008380	17,024	51.25
Hybrid Models							
CNN + LSTM	88.24	0.89	0.88	0.88	0.0003687	78,809	8.71
CNN + Transformer	89.85	0.90	0.90	0.90	0.0007675	35,313	47.22
CNN + RF	89.09	0.90	0.90	0.89	0.0085857	87,170	8.72

## Data Availability

The data presented in this study are available on request from the corresponding author.

## References

[1] Agoramoorthy M., Ali A., Sujatha D., Michael Raj T.F., Ramesh G. (2023). An Analysis of Signature-Based Components in Hybrid Intrusion Detection Systems. Proceedings of the 2023 Intelligent Computing and Control for Engineering and Business Systems (ICCEBS).

[2] Liu H., Lang B. (2019). Machine learning and deep learning methods for intrusion detection systems: A survey. Appl. Sci..

[3] Yu W., Griffith D., Ge L., Bhattarai S., Golmie N. (2015). An integrated detection system against false data injection attacks in the smart grid. Secur. Commun. Netw..

[4] Chen Z., Xu G., Mahalingam V., Ge L., Nguyen J., Yu W., Lu C. (2016). A Cloud Computing Based Network Monitoring and Threat Detection System for Critical Infrastructures. Big Data Res..

[5] Yu W., Xu G., Chen Z., Moulema P. A cloud computing based architecture for cyber security situation awareness. Proceedings of the 2013 IEEE Conference on Communications and Network Security (CNS).

[6] Yu W., Wang X., Fu X., Xuan D., Zhao W. (2009). An Invisible Localization Attack to Internet Threat Monitors. IEEE Trans. Parallel Distrib. Syst..

[7] Liang F., Hatcher W.G., Liao W., Gao W., Yu W. (2019). Machine Learning for Security and the Internet of Things: The Good, the Bad, and the Ugly. IEEE Access.

[8] Hatcher W.G., Yu W. (2018). A Survey of Deep Learning: Platforms, Applications and Emerging Research Trends. IEEE Access.

[9] Tian P., Chen Z., Yu W., Liao W. (2021). Towards asynchronous federated learning based threat detection: A DC-Adam approach. Comput. Secur..

[10] Jayalaxmi P.L.S., Saha R., Kumar G., Conti M., Kim T.H. (2022). Machine and Deep Learning Solutions for Intrusion Detection and Prevention in IoTs: A Survey. IEEE Access.

[11] Al-Garadi M.A., Mohamed A., Al-Ali A.K., Du X., Ali I., Guizani M. (2020). A Survey of Machine and Deep Learning Methods for Internet of Things (IoT) Security. IEEE Commun. Surv. Tutor..

[12] Maniriho P., Mahmood A.N., Chowdhury M.J.M. (2024). A Survey of Recent Advances in Deep Learning Models for Detecting Malware in Desktop and Mobile Platforms. ACM Comput. Surv..

[13] Wang A. (2023). Development of an IoT-Based Parking Space Management System Design. Int. J. Appl. Inf. Manag..

[14] Govindaraju S., Indirani M., Maidin S.S., Wei J. (2024). Intelligent Transportation System’s Machine Learning-Based Traffic Prediction. J. Appl. Data Sci..

[15] Liu X., Qian C., Hatcher W.G., Xu H., Liao W., Yu W. (2019). Secure Internet of Things (IoT)-Based Smart-World Critical Infrastructures: Survey, Case Study and Research Opportunities. IEEE Access.

[16] Heidari A., Jabraeil Jamali M.A. (2023). Internet of Things intrusion detection systems: A comprehensive review and future directions. Clust. Comput..

[17] Lin J., Yu W., Zhang N., Yang X., Zhang H., Zhao W. (2017). A Survey on Internet of Things: Architecture, Enabling Technologies, Security and Privacy, and Applications. IEEE Internet Things J..

[18] Hajiheidari S., Wakil K., Badri M., Navimipour N.J. (2019). Intrusion detection systems in the Internet of things: A comprehensive investigation. Comput. Netw..

[19] Saied M., Guirguis S., Madbouly M. (2025). Review of filtering based feature selection for Botnet detection in the Internet of Things. Artif. Intell. Rev..

[20] Rodríguez-Gómez R.A., Maciá-Fernández G., García-Teodoro P. (2013). Survey and taxonomy of botnet research through life-cycle. ACM Comput. Surv. (CSUR).

[21] Alrawi O., Lever C., Valakuzhy K., Snow K., Monrose F., Antonakakis M. The circle of life: A large-scale study of the IoT malware lifecycle. Proceedings of the 30th USENIX Security Symposium (USENIX Security 21).

[22] Merlino V., Allegra D. (2024). Energy-based approach for attack detection in IoT devices: A survey. Internet Things.

[23] Jung W., Zhao H., Sun M., Zhou G. (2020). IoT botnet detection via power consumption modeling. Smart Health.

[24] Jung W., Feng Y., Khan S.A., Xin C., Zhao D., Zhou G. (2022). Deepauditor: Distributed online intrusion detection system for iot devices via power side-channel auditing. Proceedings of the 2022 21st ACM/IEEE International Conference on Information Processing in Sensor Networks (IPSN).

[25] Khan S.A., Li Z., Jung W., Feng Y., Zhao D., Xin C., Zhou G. (2024). DeepShield: Lightweight privacy-preserving inference for real-time IoT botnet detection. Proceedings of the 2024 IEEE 37th International System-on-Chip Conference (SOCC).

[26] Jung W., Feng Y., Khan S.A., Xin C., Zhao D., Zhou G. (2022). Demo Abstract: A Distributed Power Side-channel Auditing System for Online loT Intrusion Detection. Proceedings of the 2022 21st ACM/IEEE International Conference on Information Processing in Sensor Networks (IPSN).

[27] Li Z., Perez B., Khan S.A., Feldhaus B., Zhao D. (2021). A new design of smart plug for real-time iot malware detection. Proceedings of the 2021 IEEE Microelectronics Design & Test Symposium (MDTS).

[28] Ma W., Wang X., Dong J., Hu M., Zhou Q. (2025). A Lightweight Method for Botnet Detection in Internet of Things Environment. IEEE Trans. Netw. Sci. Eng..

[29] Przybocki P., Vassilakis V.G. (2023). An analysis into physical and virtual power draw characteristics of embedded wireless sensor network devices under dos and rpl-based attacks. Sensors.

[30] Sabbir (2025). IoT Malware Data. https://www.kaggle.com/datasets/sa05042/iot-malware-data.

[31] Saied M., Guirguis S. (2025). Explainable artificial intelligence for botnet detection in internet of things. Sci. Rep..

[32] Kikissagbe B.R., Adda M. (2024). Machine learning-based intrusion detection methods in IoT systems: A comprehensive review. Electronics.

[33] Hayadi B.H., El Emary I.M. (2024). Enhancing Security and Efficiency in Decentralized Smart Applications through Blockchain Machine Learning Integration. J. Curr. Res. Blockchain.

[34] Prasetio A.B. (2025). Scam Detection in Metaverse Platforms Through Advanced Machine Learning Techniques. Int. J. Res. Metaverse.

[35] Pratama S.F. (2025). Fraudulent Transaction Detection in Online Systems Using Random Forest and Gradient Boosting. J. Cyber Law.

[36] Rahman M.M., Al Shakil S., Mustakim M.R. (2025). A survey on intrusion detection system in IoT networks. Cyber Secur. Appl..

[37] Neto E.C.P., Iqbal S., Buffett S., Sultana M., Taylor A. (2025). Deep learning for intrusion detection in emerging technologies: A comprehensive survey and new perspectives. Artif. Intell. Rev..

[38] Bagui S., Wang X., Bagui S. (2021). Machine Learning Based Intrusion Detection for IoT Botnet. Int. J. Mach. Learn. Comput..

[39] Idrissi I., Boukabous M., Azizi M., Moussaoui O., El Fadili H. (2021). Toward a deep learning-based intrusion detection system for IoT against botnet attacks. IAES Int. J. Artif. Intell..

[40] Abraham B., Mandya A., Bapat R., Alali F., Brown D.E., Veeraraghavan M. A Comparison of Machine Learning Approaches to Detect Botnet Traffic. Proceedings of the 2018 International Joint Conference on Neural Networks (IJCNN).

[41] Ullah S., Wu J., Lin Z., Kamal M.M., Mostafa H., Sheraz M., Chuah T.C. (2025). Comparative analysis of deep learning and traditional methods for IoT botnet detection using a multi-model framework across diverse datasets. Sci. Rep..

[42] Hossain M.A. (2025). Deep learning-based intrusion detection for IoT networks: A scalable and efficient approach. EURASIP J. Inf. Secur..

[43] Korba A.A., Diaf A., Bouchiha M.A., Ghamri-Doudane Y. (2025). Mitigating iot botnet attacks: An early-stage explainable network-based anomaly detection approach. Comput. Commun..

[44] Ding F., Li H., Luo F., Hu H., Cheng L., Xiao H., Ge R. DeepPower: Non-intrusive and deep learning-based detection of IoT malware using power side channels. Proceedings of the 15th ACM Asia Conference on Computer and Communications Security.

[45] Tekin N., Acar A., Aris A., Uluagac A.S., Gungor V.C. (2023). Energy consumption of on-device machine learning models for IoT intrusion detection. Internet Things.

[46] Campos A.D., Lemus-Prieto F., González-Sánchez J.L., Lindo A.C. (2024). Intrusion Detection for IoT Environments Through Side-Channel and Machine Learning Techniques. IEEE Access.

[47] Zhou J., Hai T., Jawawi D.N.A., Wang D., Lakshmanna K., Maddikunta P.K.R., Iwendi M. (2023). A lightweight energy consumption ensemble-based botnet detection model for IoT/6G networks. Sustain. Energy Technol. Assess..

[48] Bushehri A.S., Amirnia A., Belkhiri A., Keivanpour S., de Magalhães F.G., Nicolescu G. (2023). Deep learning-driven anomaly detection for green IoT edge networks. IEEE Trans. Green Commun. Netw..

[49] Cathis A., Li G., Wei S., Orshansky M., Tiwari M., Gerstlauer A. Sok paper: Power side-channel malware detection. Proceedings of the International Workshop on Hardware and Architectural Support for Security and Privacy 2024.

[50] Nimmy K., Dilraj M., Sankaran S., Achuthan K. (2023). Leveraging power consumption for anomaly detection on IoT devices in smart homes. J. Ambient Intell. Humaniz. Comput..

[51] Almeida A., Asif M., Rahman M.T., Rahman M.A. (2024). Side-Channel-Driven Intrusion Detection System for Mission Critical Unmanned Aerial Vehicles. Proceedings of the 2024 25th International Symposium on Quality Electronic Design (ISQED).

[52] Lightbody D., Ngo D.M., Temko A., Murphy C.C., Popovici E. (2023). Attacks on IoT: Side-channel power acquisition framework for intrusion detection. Future Internet.

[53] Lightbody D., Ngo D.M., Temko A., Murphy C.C., Popovici E. (2024). Dragon_Pi: IoT side-channel power data intrusion detection dataset and unsupervised convolutional autoencoder for intrusion detection. Future Internet.

[54] Albasir A., Naik K., Manzano R. (2023). Toward improving the security of IoT and CPS devices: An AI approach. Digit. Threat. Res. Pract..

[55] Das D., Lahkar M.P., Gogor A., Boro D. Side-Channel Analysis for Malicious Activity Detection Using Deep Learning Techniques. Proceedings of the 1st International Conference on Cognitive & Cloud Computing (IC3Com 2024).

